# First report of a p.Cys484Tyr Notch3 mutation in a CADASIL patient with acute bilateral multiple subcortical infarcts—case report and brief review

**DOI:** 10.1186/s12883-024-03573-8

**Published:** 2024-02-26

**Authors:** Weili Liu, Jie Zhang, Jian Li, Shuai Jia, Yanqiang Wang, Jianhong Geng, Yaozhen Wang

**Affiliations:** 1 Department of Neurology II, Affiliated Hospital of Weifang Medical University, School of Clinical Medicine, Weifang Medical University, Weifang, China; 2 Department of Respiratory and Critical Care Medicine, Affiliated Hospital of Weifang Medical University, School of Clinical Medicine, Weifang Medical University, Weifang, China

**Keywords:** CADASIL, Acute bilateral subcortical infarcts, NOTCH3 gene, Novel mutation

## Abstract

**Background:**

CADASIL(Cerebral autosomal dominant arteriopathy with subcortical infarcts and leukoencephalopathy)is an inherited small vessel disease caused by mutations in NOTCH3 gene. Although NOTCH3 has numerous hotspots of gene mutations, mutations in exons 9 are rare. The p.C484T gene mutation type associated with it has not been reported in any relevant cases yet. Furthermore, CADASIL patients rarely present with acute bilateral multiple subcortical infarcts.

**Case presentation:**

We report the case of a Chinese female patient with CADASIL who experienced “an acute bilateral subcortical infarction” because of“hemodynamic changes and hypercoagulability”. In genetic testing, we discovered a new Cys484Tyr mutation in exon 9, which has also been found in the patient’s two daughters.

**Conclusions:**

It is important to note that this discovery not only expands the mutation spectrum of Notch3 mutations in CADASIL patients, but also examines the mechanism behind acute bilateral subcortical infarction in CADASIL patients via case reviews and literature reviews, in order to provide some clinical recommendations for early intervention, diagnosis, and treatment in similar cases in the future.

## Background

CADASIL is one of the most common autosomal dominant cerebrovascular diseases. According to incomplete statistics, its worldwide incidence rate is approximately3-5/100,000 [1]. It is strongly associated with a mutation in the NOTCH3 gene on chromosome 19 [2]. A majority of mutations occur in exons 3,4 and 11, with a limited amount occurring in exon 9 [3]. Approximately 60 to 85% of patients with CADASIL will experience recurrent ischemic events, which are primarily characterized by unilateral lacunar infarctions, Clinical manifestations that are characteristic of acute bilateral subcortical infarctions are very rare in CADASIL.Here, we report a case with an acute bilateral subcortical infarction as the primary clinical manifestation and carrying a rare mutation of the NOTCH3 gene.

## Case report

**Fig. 1:**
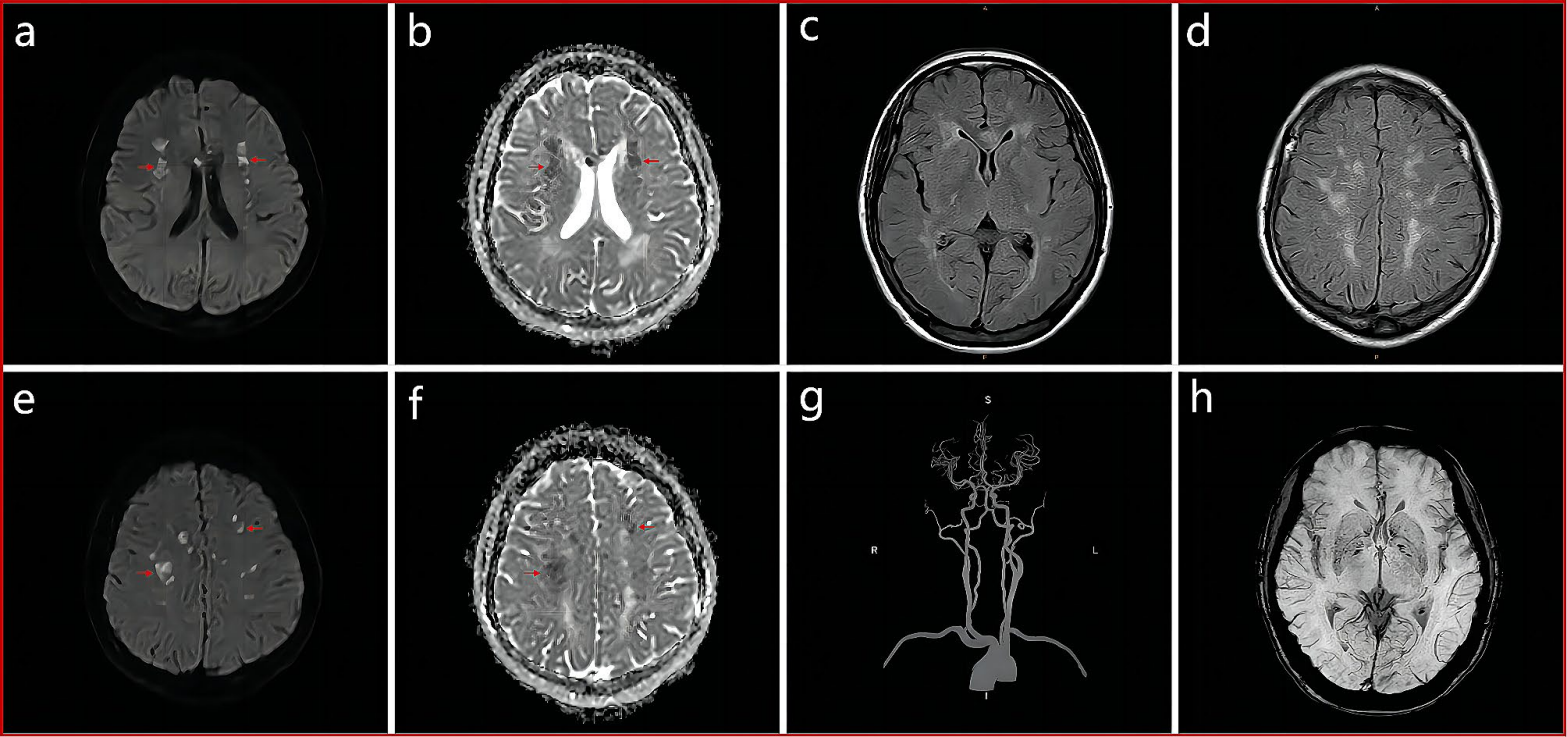
Acute bilateral multiple subcortical infarcts in patient (**a**, **b**, **e**, **f**). White matter demyelination (**c**, **d**). CT angiography (**g**). SWI (**h**)

**Fig. 2:**
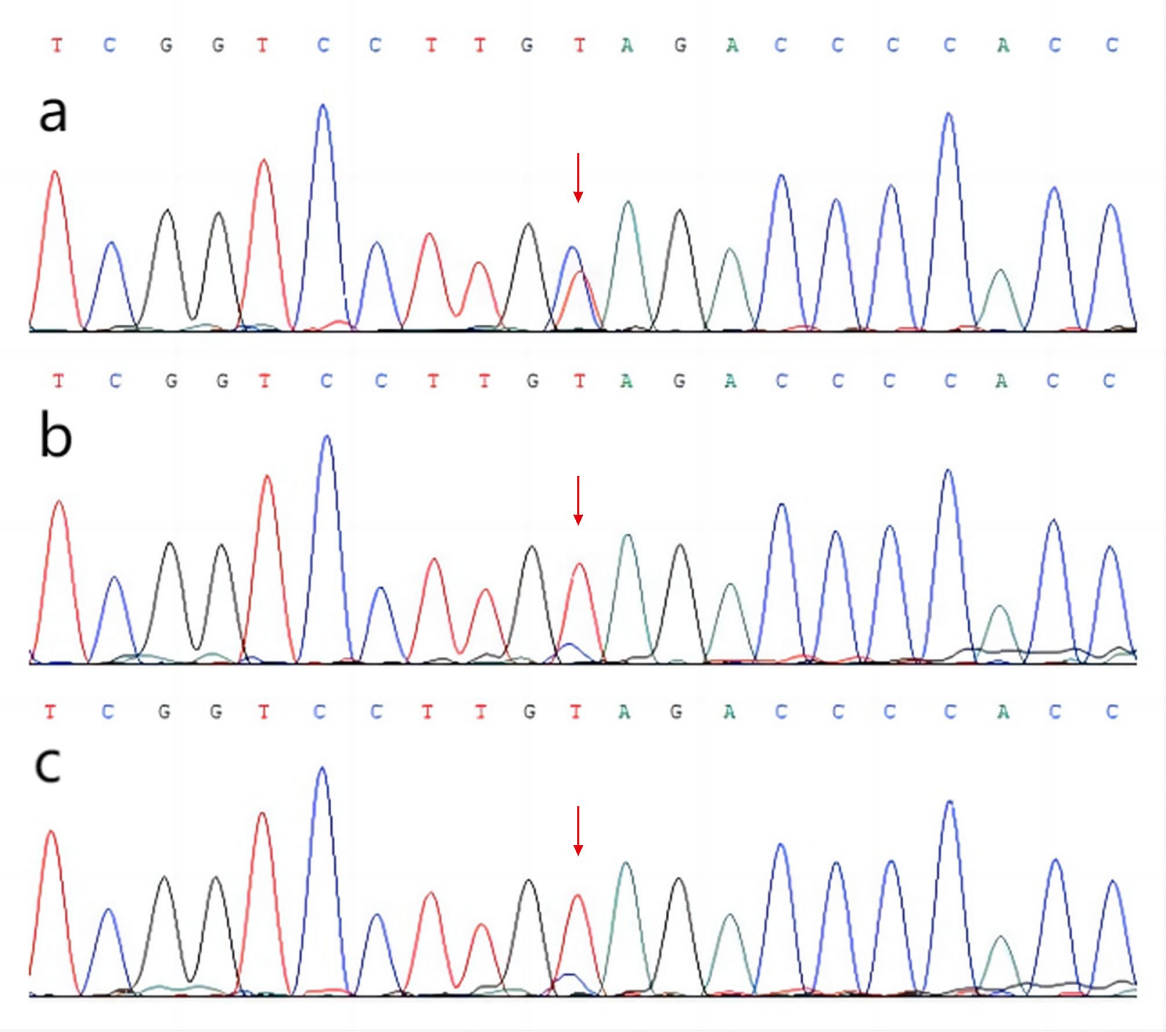
Genetic analysis: NOTCH3 gene mutation in proband (**a**) and two daughters (**b**, **c**)

A 52-year-old woman was admitted to Affiliated Hospital of Weifang Medical University’s Department of Neurology in June 2023 for “sudden speech impairment with left limb dysfunction for two days and aggravation for a day“. Past history: Recurrent dizziness started 30 years ago, Disc herniation surgery two weeks ago. Family background: Her mother died at the age of 70 after a cerebral infarction, her younger brother has leukemia, and her father, younger sister, husband, and two daughters are in good health. Physical examination of the nervous system: The patient had clear consciousness, motor aphasia, left central facial paralysis, left limb muscle strength of 4. Diagnosis procedures: At the time of admission, a cranial magnetic resonance imaging (MRI) revealed multiple fresh cerebral infarctions in bilateral subcortical regions. The patient had a low fever during hospitalization. The diagnosis of CADASIL was confirmed by relevant testing and examinations to rule out immune, infectious, tumor, genetic, and metabolic causes. Inspection of laboratory indexes: electrolytes, liver function, kidney function, blood glucose, urine routine, myocardial enzyme spectrum, blood homocysteine, procalcitonin, IL-2 + IL-4 + IL-10 +IFN-γ+TNF-α, blood culture, two items of rheumatism, three items of antiphospholipid antibodies, preoperative infection indicators, antinuclear antibody spectrum, antivasculitis antibody spectrum, CA125, HE4, CEA, AFP, CA19-9 did not reveal any significant abnormalities. The blood routine + CRP + SAA (upon admission): CRP 43.85 mg/L, WBC 15.87 10^9/L, neutrophil percentage 86.8%, hemoglobin 113 g/L, platelet count 410 10^9/L, SAA 198.01 mg/L. Fibrinogen 6.93 g/L, D-dimer 0.89 mg/L,Triglycerides 2.85mmol/L, IL-6 15.36pg/ml.Auxiliary examination: CT angiography (Fig. 1g), TCD foam test, cardiac color ultrasound, bilateral lower limb vein color ultrasound, 24-hour dynamic electrocardiogram, dynamic electroencephalogram, and chest CT revealed no significant abnormal images. CADASIL scores 13 points, MMSE scores 28 points, and Moca scores 27 points. Head MRI/DWI/SWI: 1. Multiple fresh cerebral infarctions in bilateral frontal lobes, bilateral paraventricular trigones, bilateral radiating coronal areas, and bilateral corpus callosum knees (Fig. 1a, b, e, f); 2. Multiple lacunar cerebral infarctions with partial softening over bilateral frontal, parietal, and temporal lobes, coronal radiation, basal ganglia, and left cerebellum; White matter demyelination around bilateral ventricles (Fig. 1c, d); 3. SWI: normal (Fig. 1h). Genetic analysis: NOTCH3 gene mutation in proband (Fig. 2a) and two daughters (Fig. 2b, c): c.1451 G > A (p. Cys484Tyr).Upon admission to the hospital, the patient received aspirin, statins, and treatments for circulation, nutrition, and nerves.Following discharge from the hospital, the patient’s symptoms disappeared.We followed up with the patients three and six months after discharge, and there were no other symptoms except occasional episodic dizziness.

## Discussion

CADASIL is an inherited disease that affects small vessels in adults. Over 400 mutation types have been identified since 1996 when the Notch3 gene was discovered as its pathogenic gene [4]. Most gene mutations result from missense mutations (95%), whereas intraframe deletions, frameshift deletions, and splice site mutations are relatively uncommon. It is common for mutations to result in an odd number of cysteine residues, which disrupts normal disulfide bond formation, resulting in Notch3 aggregate formation [5].Most gene mutations are located in exons 2–24, which encode 34 EGFR, particularly in exons 3, 4, 5, 6, 8, 11, and 18 [6]. The number and distribution of gene mutations in different populations and regions has been extensively reported. The gene mutation hotspots in Europe are concentrated in exons 2–6, with exon 4 having the highest frequency (this pattern is more prevalent in France, the United Kingdom, and Germany). In the Asian population, mutation hotspots are mostly in exon 11 (40–85%) and exon 4 (20–40%), which is common in South Korea and China. According to a study involving 1401 families of CADASIL, the most commonly mutated genes among Asians are p.R544C and p.R607C, while among Caucasians they are p.R1006C and p.R141C [4].In recent years, due to the maturity and popularization of genetic testing technology, more and more new types of gene mutations in CADASIL have been reported.Here, we provide a brief overview of the new types of genetic mutations in CADASIL that have been reported in various places in the past 10 years(Table 1).According to our analysis, the most new types of mutations reported in CADASIL in recent years came from Asia, mainly from China; more gene mutation hotspots: exon 3,4,6,24, more common amino acid changes: Cys variations Tyr, Ser, and Gly. So far,there have been very few reports of gene-related mutations in exon 9.Among them,Dr.Gido [2] not only identified a specific gene mutation type p.G498C that induces exon 9 hopping, but also provided the first human evidence to show that cysteine corrective NOTCH3 exon skipping is associated with only minimal vascular NOTCH3 aggregation and a relatively mild later-onset phenotype.In our patient, a heterozygous missense mutation in the C484T gene was located in exon 9. It has never been showed in the East Asian Population Database or CADASIL case reports. Additionally, the patient’s two daughters were also found to have the same genetic mutation. Through further research and follow-up of the entire family, this discovery not only complements the rare gene mutation in exon 9, but may also provide more clinical data for patients.

**Table 1:** New NOTCH3 mutations in CADASIL from 2014 to 2024

Continent	Country	Exon	Nucleotide change	Amino acid change
Asia	China	2	c.128G > C	p.Cys43Ser
		3	c.218G > C c.331G > T c.316T > G	p.Gly73Ala p.Gly111Cys p.Cys106Gly
		4	c.635G > C c.446G > T	p.Cys212Ser p.Gly149Val
		6	c.931T > G c.1013G > C	p.Cys311Gly p.Cys338Ser
		13	c.1594 C > T	p.Pro652Ser
		19	c.3043T > A	p.Cys1015Ser
		20	c.3299G > A	p.Arg1100His
		24	c.3892T > G	p.Cys1298Gly
		29	c.5282G > A	p.Arg1761His
	Japan	24	c.3879 C > G	p.Cys1293Trp
	Korean	4	c.499 C > T	p.Pro167Ser
	Saudi Arabia	19	c.3009G > T	p.Trp1003Cys
Europe	Russia	1	c.208G > T	p.Gly70Cys
		7	c.1136G > A	p.Cys379Tyr
		10	c.1547G > A	p.Cys516Tyr
	Spain	3	c.244T > C	p.Cys82Arg
	Italy	24	c.3944G > A	p.Cys1315Tyr
North America	Canada	8	c.1337G > A	p.Cys446Tyr

CADASIL is characterized by ischemic strokes or transient ischemicattacks, progressive dementia and migraines and no risk factors for cerebrovascular disease like hypertension, diabetes, smoking, drinking, etc [6]. The most common imaging manifestations include high white matter signal intensity, lacunar infarctions, and cerebral microbleeds, most commonly involved with the external capsule, lateral ventricle, and temporal pole [7]. The clinical presentation of this patient may be described as “atypical lacunar syndrome”.It is a rare clinical presentation.According to Professor Arboix’s study, they only accounted for 6.8% of the lacunar strokes in a sample of 39 patients with atypical lacunar syndromes [8].Despite the fact that ischemic cerebrovascular events are the most common clinical manifestation, there have been less than 20 reports of cases characterized by “acute bilateral subcortical infarction” as the main clinical manifestation. The rarity of this clinical manifestation makes it highly susceptible to misdiagnosis. The diagnosis process was also time-consuming, especially given that we spent some time excluding rheumatism, immunity, infection, and tumors as possible causes. CADASIL lesions mainly affect small arteries; under optical microscopy, vascular smooth muscle cells appeardegenerated and absent, resulting in fibrosis and thickening of the vascular walls. Ultrastructure shows the deposition of granular electron dense osmiophilic substances at the basement membrane depression of vascular smooth muscle cells. Currently, there is no consensus on the mechanism of acute bilateral subcortical infarction in CADASIL patients. Several studies have shown that extensive small vessel lesions cause the brain of CADASIL patients to be in a state of long-term hypoperfusion and significantly decrease their ability to regulate blood flow fluctuations, which may be an important reason [9, 10]. It is also possible that hypercoagulability in the body is a factor. Alessandro’s work suggests that the hypercoagulable state after infection with novel coronavirus may contribute to acute bilateral multiple cerebral infarction in CADASIL patients [11]. The case we reported had undergone surgery 2 weeks before the onset of the disease. Upon admission, she was anemic, had elevated fibrinogen levels, and a low fever and blood pressure ranging from 90 to 100/60-70mmHg over the course of the illness. This acute bilateral ischemic event may have been caused by hypercoagulability and changes in hemodynamics. Therefore, Reducing the occurrence of the above risk factors may reduce the probability of acute cerebral infarction in CADASIL patients. Besides, patients may benefit from personalized treatment for these risk factors.

Of course, there are many limitations to our research.First of all,because of the patient’s personal wishes and limited clinical information on relatives, we did not perform skin biopsies and were unable to establish a complete pedigree.As a result of their short discharge time, the patient and their daughters currently receive a relatively short follow-up, and we are looking forward to more comprehensive follow-up in the future.Additionally, the limitations of our search methods and thinking methods have prevented us from providing a more comprehensive review of recent gene mutations found in CADASIL. Despite the many limitations of our study, we must acknowledge, based on the review of this case and the extensive literature search, that CADASIL’s omics studies have been instrumental in understanding the biological mechanisms, neuroimaging or clinical manifestations of the disease, and may also help identify potential drug targets [12].

## Conclusion

In summary, We reported a new gene mutation type c.1451 G > A (p. Cys484Tyr) and briefly reviewed the distribution and pathogenicity of Notch3 gene mutation hotspots, to provide some molecular biology data for CADASIL. Although acute bilateral cerebral infarction is a rare clinical manifestation of CADASIL, it is important to consider the possibility of CADASIL when encountering similar patients without clear risk factors for cerebral hemorrhage. Moreover, we hope to provide new clinical ideas for early intervention and diagnosis and treatment of similar patients by discussing the pathogenesis of acute bilateral subcortical infarction in CADASIL patients.Finally, we’d like to invite more researchers to explore and study CADASIL genomics, gene editing, pathogenesis and individualized treatment.

## Data Availability

The data that support the findings of this study are available from the corresponding author upon reasonable request.

## Abbreviations

CADASIL: Cerebral autosomal dominant arteriopathy with subcortical infarcts and leukoencephalopathy
CRP: C-reactive protein
MRI: Magnetic resonance imaging
TCD: Transcranial Doppler
DWI: Diffusion weighted imaging
MoCA: Montreal Cognitive Assessment
CT: computed tomography
MMSE: mini-mental state examiniation
SWI: Susceptibility weighted imaging
